# Injury severity data for front and second row passengers in frontal crashes

**DOI:** 10.1016/j.dib.2016.03.046

**Published:** 2016-03-17

**Authors:** Theresa Atkinson, Leszek Gawarecki, Massoud Tavakoli

**Affiliations:** Kettering University, USA

## Abstract

The data contained here were obtained from the National Highway Transportation Safety Administration׳s National Automotive Sampling System – Crashworthiness Data System (NASS-CDS) for the years 2008–2014. This publically available data set monitors motor vehicle crashes in the United States, using a stratified random sample frame, resulting in information on approximately 5000 crashes each year that can be utilized to create national estimates for crashes. The NASS-CDS data sets document vehicle, crash, and occupant factors. These data can be utilized to examine public health, law enforcement, roadway planning, and vehicle design issues. The data provided in this brief are a subset of crash events and occupants. The crashes provided are exclusively frontal crashes. Within these crashes, only restrained occupants who were seated in the right front seat position or the second row outboard seat positions were included. The front row and second row data sets were utilized to construct occupant pairs crashes where both a right front seat occupant and a second row occupant were available. Both unpaired and paired data sets are provided in this brief.

**Specifications Table**

**Table:** 

Subject area	*Biology*
More specific subject area	*Vehicle Occupant Safety*
Type of data	*Tables, data files*
How data was acquired	*Accessed from the National Highway Transportation Safety Administration website*
Data format	*Raw coded data*
Experimental factors	*A segregated sample of frontal crashes involving passenger vehicles with second row occupants was extracted from crashes documented in the National Automotive Sampling System – Crashworthiness Data System, data years 2008–2014*
Experimental features	*Injury risk analysis for groups of passengers was performed using the Surveyfreq and Surveylogistic functions in SAS to examine injury risk while accounting for sample design and potential confounding variables*
Data source location	*United States*
Data accessibility	*Data is within in the article*

**Value of the data**

• Occupant characteristics and injury severity (assessed using injury scores, hospital stay or work days lost) can be examined for restrained occupants and national estimates for outcomes estimated using the NASS-CDS sample design and weights.
• Vehicle and seating information can be examined in light of occupant age and physical characteristics.
• Occupant injury for subsets of frontal crash cases involving specific types of vehicles, specific crash force orientation or objects impacted may be examined.
• The paired data set describes occupants who are seated in the same vehicle during a frontal crash. These data may provide insight into social factors that contribute to injury risk. For example, the data provide age ranges for typical co-occupants within a motor vehicle environment which might be of interest in the construction of safety messages aimed at young drivers.
• The tabulated data estimates injury rates for occupants by seat position and age. These may provide law enforcement and other agencies baseline injury data they may use to compare to their local data.

## 1 Data

The data contained here were obtained from NHTSA׳s ftp portal for the National Automotive Sampling System – Crashworthiness Data System [1], which documents crash conditions and occupant injuries for crashes occurring in the United States. These data are obtained through a sampling framework that is described in the data set and supporting documentation [2]. Utilizing the sampling frame and weights one can obtain national estimates for frequency for various motor vehicle crash related events. Only those occupants seated in the right front seat position (seat position #13) and those who were seated in the outboard positions of the second row (seat positions 21 and 23), who were indicated to have been using both a lap and shoulder belt by the NASS crash investigator are included here. The data in the raw files is coded. The codes are documented in files located on the NHTSA website [3].

## 2 Experimental design, materials and methods

Frontal crash cases were segregated from crashes documented in the NASS-CDS data sets for the years 2008–2014. The General Vehicle data file was utilized in conjunction with the External Vehicle file for each year. The files were joined using the primary sampling unit, case identifier, and vehicle number codes. The resulting data file was filtered to select only those vehicle that were indicated to have experienced a primary crash force direction corresponding to 11:00–1:00, where 12:00 would indicate an impact force directed perpendicular to the vehicle׳s front (NASS variable identifier “direction of force” (DOF1=F)). Within these frontal force cases, only those vehicles that exhibited a primarily “frontal” damage field (NASS variable identifier “general area of damage” (GAD1)) were included. Any vehicle that experienced a rollover was excluded. Further, any vehicle with a model year before 2000 was excluded. Lastly the data was filtered to include only sedans (2 door coupe – wagons), Sport Utility Vehicles, minivans and trucks were included in the data set (vehicle type codes: 2–6, 14–16, 21–21, 30–31).

The vehicle and crash data were joined to the Occupant data file. Occupant physical characteristics, overall injury severity, seat position (i.e. seat back angle), and other occupant specific data were obtained. The occupants included in the files provided in this brief are the right front seat position (seat code 13) and the outboard seat positions in the second row (seat codes 21 and 23). Only occupants who were 13 years or older, who were also utilizing the lap and shoulder belts, were retained in the final data set. Each row of these data files describes a single occupant and the vehicle/crash they experienced. The file for the right front row occupants contains 1771 entries that represent approximately 661,000 persons involved in motor vehicle crashes. The second row occupant file contains 436 entries that represent approximately 132,000 persons.

Injury rates in front row seated occupants were higher than those for second row seated occupants (Table 1). The “MAIS” is the maximum abbreviated injury score, where scores rate fatality risk and range from 1 (scratch or bruise) to 6 (unsurvivable). The “ISS” is the injury severity score which utilizes up to 3 injury scores for an occupant. The ISS level of 9 was suggested by Palmer [4] as a threshold for serious injury.

Subsets of older occupants composed of only those in vehicles that were produced in 2006 or later were compared (Table 2). In these cases the older occupants in the second row exhibited an injury rate that was higher than that in the front right occupants. However, the injury rates were obtained considering all crashes, which included many non-injurious crashes. In addition, these occupants were not necessarily exposed to the same crash, as they were not occupant pairs. Atkinson et al.’s [5] analysis showed a higher injury rate in front row seated occupants when non-injurious crashes were removed from the data sets.

The source files for these data sets are provided online in the Supplementary Material.

**Fig. 1:**
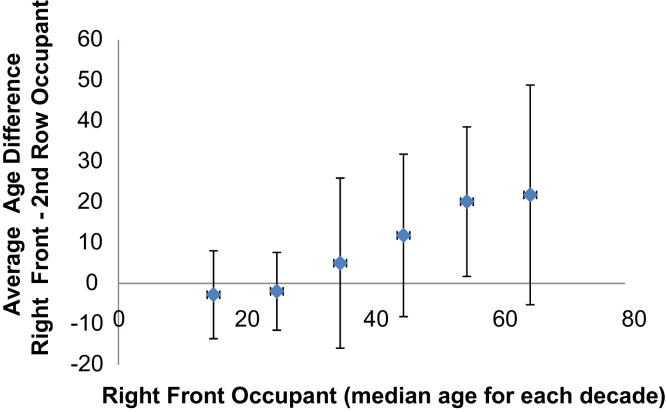
The average age difference between paired occupants (central dot) and ±1 standard deviation (whiskers), where data was grouped by the age of the right front occupant. The apparent linear trend indicated by the averages was not supported by regression analysis as the groups involving right front occupants ≤40 years have much higher numbers of cases.

A second data set consisting of occupant pairs was created by joining the right front occupant to a second row (outboard seated) occupant in the same vehicle. Where there was more than one second row occupant available a second pair was generated. Each line of the data file represents a pair of occupants. The occupant seated in the second row is listed first along with any occupant specific data (each denoted as 2nd_row_ data field) and the data associated with the matched front right seat occupant is listed second (each denoted as front rt_rest _data field). The paired data file includes 238 pairs. Fig. 1.

The source file for the paired data set is provided online in the Supplementary Material.

The paired data was reduced to those pairs that were at most 15 years different in age. The crash and occupant factors suspected to play a role in moderating the likelihood of injury were examined using logistic regression (Surveylogistic procedure, SAS Institute). The specific outcome studied was the event of a second row seated occupant having an injury severity exceeding that of the paired right front occupant (Table 3).

## Figures and Tables

**Table 1:** Percent of restrained occupants (age ≥13 years) in frontal crashes (not pairs) grouped as indicated on the left, with injuries at the levels indicated across the top. Values are reported as percent at the injury level indicated at the top of the column and 95th confidence interval on the percent, based on the weighted data and sample design, obtained using SAS Surveyfreq procedure.

	**% MAIS≥2 [95th CI]**	**% MAIS≥3 [95th CI]**	**% ISS>9 [95th CI]**
**All Front Rt**^a^**Occupants**	5.1 [3.0–7.1]	1.6 [0.7–2.6]	1.5 [0.6–2.5]
**All 2nd Row**^b^**Occupants**	2.7 [0.9–4.6]	0.9 [0.0–1.8]	0.9 [0.0–1.8]
**Front, Age ≥25 yrs**	6.6 [3.1–10.0]	2.3 [0.7–3.8]	2.1 [0.6–3.6]
**2nd Row**^b^, **Age ≥25 yrs**	4.9 [0.0–9.9]	1.4 [0.0–3.6]	1.4 [0.0–3.6]

a Front right seat.

b Outboard seat positions.

**Table 2:** Percent of restrained adult occupants (age ≥25 years) in frontal crashes who sustained an injury severity score (ISS) greater than 9, for the conditions noted in the left column.

	**% ISS >9 [95th CI]**

**Front, Age ≥25 Yrs**	
**Vehicle MY**^a^**<׳06**	2.2 [1.0–3.4]
**Vehicle MY ≥׳06**	2.1 [0.0–4.3]
**2nd Row**^b^**, Age ≥25 yrs**	
**Vehicle MY <׳06**	5.1 [2.4–7.9]
**Vehicle MY ≥׳06**	7.9 [0.5–15.3]
**Front, Age ≥25 yrs**	
**Crash Velocity ≥30** **km/h**	6.7 [3.3–10.1]
**2nd Row**^b^**, age≥25 yrs**	
**Crash Velocity ≥30** **km/h**	9.2 [4.1–14.3]

a Model/year.

b Outboard seat positions.

**Table 3:** Odds ratios [95th confidence interval] and *p* values from the stepwise regression analysis. Not all data elements were available for all cases included in the analysis, yielding different numbers of cases included in each analysis step. The reduced model including only delta *V* (difference in vehicles’ velocities) and age difference resulted in a significant fit as indicated by p value of <0.001 in a likelihood ratio test, a high receiver operating characteristic (ROC), and as fit to the largest data set. They symbol *Δ* denotes difference (i.e. front−second row).

**Variable**	**Step 1 Odds Ratio Wald chi-sq**	**Step 2**	**Step3**	**Step 4**	**Step 5**	**Step 6**
**Model year**	0.13[0.0–8.9] *p*=0.34					
**Body Type**	0.23[0.01–4.2] *p*=0.32	0.56 [0.23–1.36] *p*=0.20	0.82[0.481.41] *p*=0.48			
**Curb weight**	1.14 [0.87–1.48] *p*=0.34	1.05[0.95–1.53] *p*=0.36				
**Delta V**	1.30 [0.95– 1.78] *p*=0.10	1.21[0.95–1.55] *p*=0.12	1.14[0.95–1.37] *p*=0.15	1.09[0.98–1.21] *p*=0.12	1.08[0.99–1.18] *p*=0.10	1.104[1.001–1.216] *p*=0.0473
**Age***Δ*	0.60 [0.27–1.35] *p*=0.22	0.67[0.40–1.12] *p*=0.13	0.79[0.59–1.06] *p*=0.11	0.86[0.74–1.01] *p*=0.07	0.91[0.83–.99] p=0.03	0.934 [0.883–0.988] *p*=0.0175
**Height***Δ*	79 [0.54–1.17] *p*=0.24	0.79[0.53–1.16] *p*=0.23	0.86[0.66–1.08] *p*=0.18	0.90[0.76–1.08] *p*=0.28		
**Weight***Δ*	1.18 [0.91–1.54] *p*=0.21	1.14[0.96–1.34] *p*=0.13	1.08[0.94–1.25] *p*=0.29	1.05[0.99–1.11] *p*=0.11	1.03[0.98–1.08] *p*=0.22	
**Model ROC**	0.944	0.889	0.863	0.838	0.841	0.864
**Number of Cases**	2935	2935	2935	2935	2935	4058
